# The role of JAK-STAT signaling pathway and its regulators in the fate of T helper cells

**DOI:** 10.1186/s12964-017-0177-y

**Published:** 2017-06-21

**Authors:** Farhad Seif, Majid Khoshmirsafa, Hossein Aazami, Monireh Mohsenzadegan, Gholamreza Sedighi, Mohammadali Bahar

**Affiliations:** 1 ENT and Head and Neck Research Center and Department, Hazrat Rasoul Akram Hospital, Iran University of Medical Sciences, Tehran, Iran; 2 Department of immunology, school of medicine, Iran University of Medical Sciences, Tehran, Iran; 3 Department of Medical Laboratory Science, Faculty of Allied Medical Sciences, Iran University of Medical Sciences, Tehran, Iran

## Abstract

The Janus kinase (JAK)-signal transducer and activator of transcription (STAT) pathway plays critical roles in orchestrating of immune system, especially cytokine receptors and they can modulate the polarization of T helper cells. This pathway is regulated by an array of regulator proteins, including Suppressors of Cytokine Signaling (SOCS), Protein Inhibitors of Activated STATs (PIAS) and Protein Tyrosine Phosphatases (PTPs) determining the initiation, duration and termination of the signaling cascades. Dysregulation of the JAK-STAT pathway in T helper cells may result in various immune disorders. In this review, we represent how the JAK-STAT pathway is generally regulated and then in Th cell subsets in more detail. Finally, we introduce novel targeted strategies as promising therapeutic approaches in the treatment of immune disorders. Studies are ongoing for identifying the other regulators of the JAK-STAT pathway and designing innovative therapeutic strategies. Therefore, further investigation is needed.

## Background

There is a reciprocal interaction between external actions and internal reactions that enables a cell to live. Each receptor like a sentinel senses stimuli and starts to transfer corps of signals to the castle of the nucleus in order to provoke vital responses. The result of this process may be proliferation, differentiation (polarization), activation/inhibition and survival/apoptosis. The **Janus kinase-signal transducer and activator of transcription (JAK-STAT)** pathway plays a major role in transferring of signals from cell-membrane receptors to the nucleus [1]. The JAK-STAT pathway is essential for a wide range of cytokines and growth factors, leading to critical cellular events, such as hematopoiesis, lactation and development of the immune system and mammary glands [2–4]. Cytokines are one of the major players of myeloid and lymphoid lineages which their receptors utilize this pathway [5]. A main subgroup of cytokines, ranging from over 60 factors, binds to receptors termed type I and type II cytokine receptors. These cytokines are inevitable for initiating and orchestrating of innate and adaptive immunities [6, 7].

**Fig. 1:**
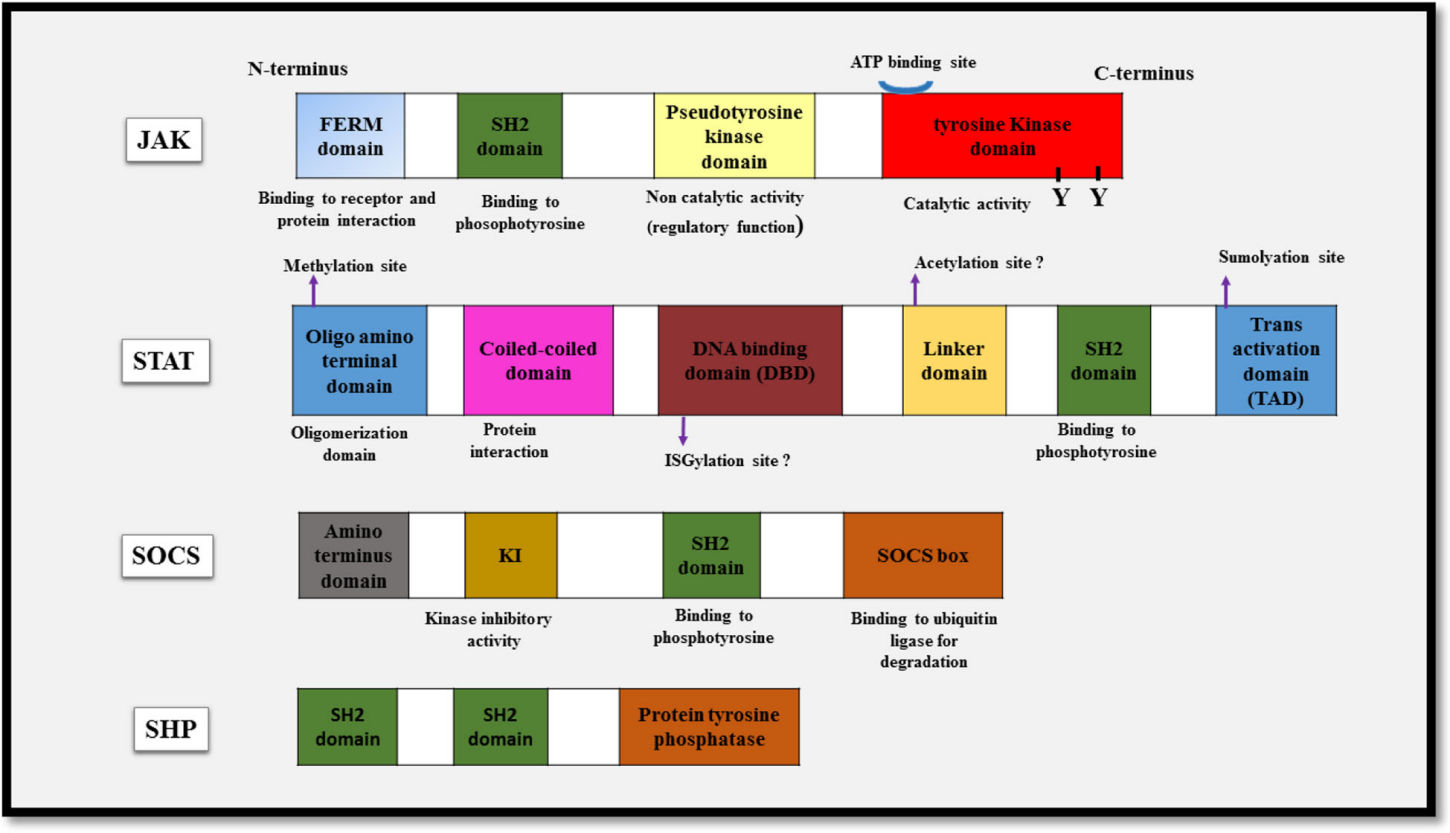
Schematic representation of the components of the JAK-STAT pathway. Each domain has been depicted and its function has been explained

**Fig. 2:**
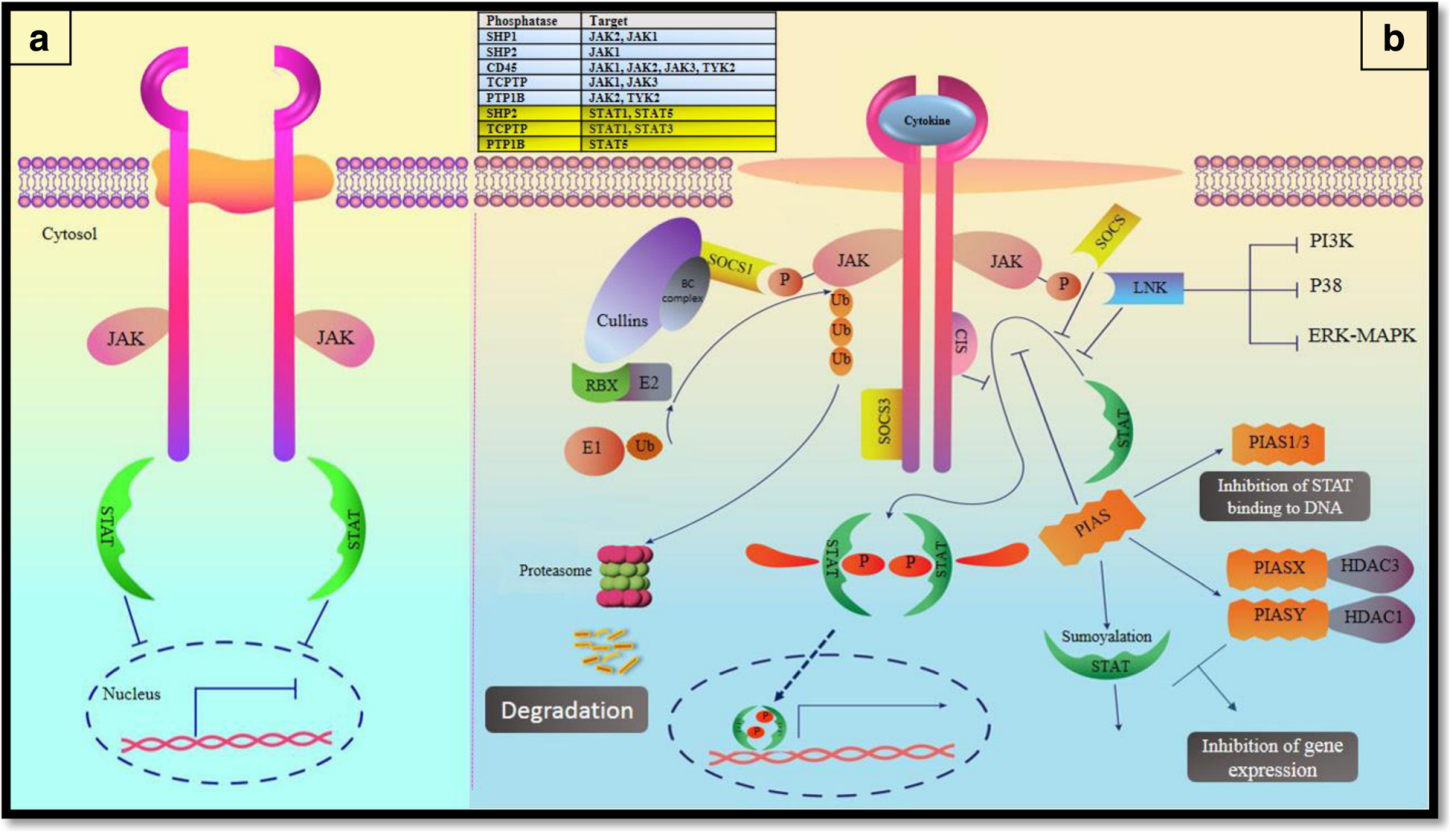
**a** The Janus kinase (JAK)- signal transducer and activator of transcription (STAT) pathway. JAKs are activated upon cytokine stimulation and phosphorylate STATs which results in dimerization and translocation of STATs to the nucleus in order to activate or suppress the transcription of genes. **b** Regulation of the JAK–STAT pathway. A schematic of negative regulators that modulate the JAK-STAT pathway. Negative regulators consist of suppressor of cytokine signaling (SOCS) proteins, Protein Inhibitors of Activated STATs (PIAS), protein tyrosine phosphatases (PTPs), such as SRC homology 2 (SH2)-domain-containing PTP1 (SHP1), SHP2, CD45, LNK, T-cell PTP (TCPTP) and protein-tyrosine phosphatase 1B (PTP1B)

**Fig. 3:**
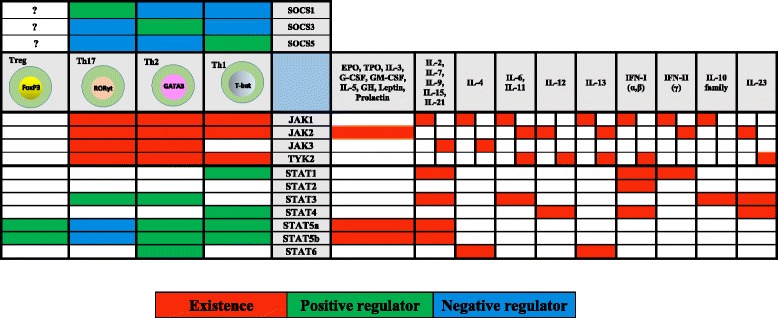
The relationship of the cytokines and JAK-STAT pathway is described. Additionally, the role of JAKs, STATs and SOCSs are displayed in Th cell differentiation

JAKs are kinds of tyrosine kinases that are bound to the cytoplasmic regions of type I and II cytokine receptors. Multimerization of receptors occurs when ligands bind to their receptors. Subunits of some receptor are expressed as homodimers, e.g. erythropoietin and growth hormone; while other receptor subunits are expressed as heteromultimers, such as interferons (IFN) and Interleukins (IL) [8]. Activation of the receptors that are associated with JAKs is critical to initiate the JAK transphosphorylation and subsequent recruitment of one or more STATs to be phosphorylated [9]. Eventually, dimerized STATs enter to the nucleus and regulate transcription of myriad target genes [8]. In this review, we aim to introduce the JAK-STAT pathway and its regulator proteins and elucidate the role of them in the differentiation of T helper(Th) cell subsets. Eventually, novel and approved therapeutic strategies are discussed in order to target the JAK-STAT pathway and its regulators in several immune disorders.

### JAK-STAT pathway

**STATs** were firstly found in 1988 as proteins that bind to interferon (IFN)-stimulated response elements of DNA sequences to stimulate the transcription of type I IFNs [10]. Then, **JAKs** were discovered in 1992 by three separate labs and the JAK-STAT pathway was coined [11].

The name of the JAK comes from a Roman two-faced god that implies two domains, including a catalytic domain and a kinase-like domain. Type I- and II receptors are constitutively associated with JAKs [12]. The binding of ligand (cytokine) to its receptor causes receptor dimerization and subsequently, JAKs are activated following close proximity [8]. These activated JAKs initiate trans-phosphorylation on specific tyrosine residues (also named transactivation), generating docking sites for recruitment of latent cytoplasmic transcription factors known as STATs [13]. Phosphorylation is the most common modification in the cell biology, which plays a crucial role in the regulation a multitude of signaling pathways [14]. Unphosphorylated STATs (Off) reside in the cytoplasm. If phosphorylation of STATs (On) and STAT dimerization occur upon activation of JAKs [9], phosphorylated STATs abandon docking sites on the receptors. Therefore, they translocate to the nucleus and bind to specific DNA sequences either to activate or suppress gene transcription [13, 15]. Although tyrosine phosphorylation of STATs has been well established, less has been reported about the effect of serine phosphorylation of STATs. It has been postulated that serine phosphorylation of STATs doesn’t depend on their tyrosine phosphorylation [16]. Additionally, several studies have indicated that serine phosphorylation of STAT1 appears to augment its transcriptional potency [16, 17], whereas serine phosphorylation of STAT3 has been reported to regulate the tyrosine phosphorylation of this molecule in a negative manner. Several serine kinases have been reported to be involved in the serine phosphorylation of STATs, including p38, Erk and JNK [18]. The JAK-STAT pathway also facilitates various cellular reactions to diverse forms of cellular stress, including hypoxia/reperfusion, endotoxin, ultraviolet light, and hyperosmolarity [19].

Human JAK family contains four JAKs: JAK1, JAK2, JAK3 and TYK2 [20]. Each JAK member comprises several distinct domains which are described as follows: **N-terminal FERM domain** was known after the discovery of proteins that contain this domain (band 4.1, Ezrin, Radixin and Moesin). FERM domain consists of three subdomains F1, F2 &F3, which are structurally similar to Ubiquitin, CoA binding and pleckstrin homology-phosphotyrosine binding domains, respectively [21]. It is responsible for protein-protein interactions, such as adaptor and scaffolding interactions with membrane associated proteins [22]. The **SH2(Src homology 2) domain** is a motif containing approximately 100 residues that binds to phosphotyrosine residues The role of SH2 domain is the activation and dimerization of STATs [23, 24]. Central **pseudokinase** domain is named because of its homology to Protein Tyrosine Kinases (PTK) domain; however, it lacks of catalytic function and appears to have a regulatory role [20, 25]. Finally, a **conserved PTK domain** is located at the C-terminus (Fig. 1). It contains approximately 250–300 residues and an ATP-binding site juxtaposing a catalytic region. It is responsible for phosphorylation of specific tyrosine residues positioned on special downstream substrates [26]. Human STAT family contains seven STATs: STAT1, STAT2, STAT3, STAT4, STAT5A, STAT5B and STAT6 [27]. They have extreme homology in the following regions: **Unique N-terminus region** involves in STAT regulation e.g. tyrosine dephosphorylation or STAT interactions, such as tetramer formation [28–30]. Then, **coiled-coil domain** which is involved in protein-protein interactions and nuclear export. The **DNA-binding domain** contains a S-type immunoglobulin fold and is also found in p53. It facilitates sequence-specific binding [31, 32]. This region recognizes TTCN3–4GAAA sequence found in the promoters of target genes [4, 27]. Finally, C-terminus region which is also called **Trans-Activation Domain (TAD)** and contains a highly conserved tyrosine residue (Fig. 1) [28]. However, several discovered varieties are involved in recruitment of special proteins, such as histone deacetylases, DNA polymerase II, etc. [31]. JAKs are activated upon cytokine attachment and intracellular region of the receptor is phosphorylated as a docking site for STATs to be recruited and phosphorylated [16]. Subsequently, STATs start hetero- or homo-dimerization through SH2 domains. There is a protein named **StIP** (STAT Interacting Protein) which is associated with JAK-STAT pathway. It seems to be as a scaffold facilitating phosphorylation of unphosphorylated STATs. Phosphorylated STATs are translocated to the nucleus in importin α-5 dependent manner via Ran nuclear import pathway. At the end of their import, dimerized STATs bind to specific DNA sequences in order to regulate transcription of their own target genes [8]. Although great information is available about the process of STAT phosphorylation, STAT dephosphorylation in the nucleus is still poorly defined [33].

### The role of JAK-STAT pathway in the regulation of Th cells

JAK-STAT pathway is provoked in two ways:

**1. Canonical pathway**: It is activated by receptor binding to the ligand and are inactivated by negative regulators such as SOCS and SHPs. It is a conventional pathway that other signaling molecules are involved in, including PI3 kinase (PI3K), Mitogen Activate Kinase (MAPK), Extracellular Receptor Kinase (ERK) [34].

**Noncanonical pathway**: In contrast to the canonical pathway, in which unphosphorylated STATs are localized in the cytoplasm, in the noncanonical pathway, a number of the unphosphorylated STATs are localized on heterochromatin in the nucleus in association with special proteins that contribute to the maintenance of heterochromatin state. Whenever STAT phosphorylation is increased (by JAK or other tyrosine kinases) the amount of unphosphorylated STATs are reduced on heterochromatin. Dispersed phosphorylated STAT bind to cognitive sites in euchromatin to induce gene expression. In fact, it can affect total transcriptional events through chromatin modification [34].

Although the mechanisms by which JAK/STAT pathway is regulated theoretically, look simple; the bimolecular consequences of signaling activation are complicated. Because numerous interactions may occur with other signaling pathways and molecules (crosstalk) [34]. JAK-STAT pathway has a critical role in the fate of T helper cells (Th). CD4^+^ Th cells can differentiate into multiple effector subsets, including Th1, Th2, Th17, regulatory T cells (Tregs) and so on (Fig. 3) [35].

- **Th1 cells** involve in the elimination of intracellular pathogens and their hallmark cytokine is IFN-γ (activating of cell mediated immunity). Th1 specific transcription factor is **T-bet** [36] STAT1 is critical for IFN-γ signaling and STAT4 is necessary for IL-12 signaling. Both of STAT1/STAT4 are key factors for Th1 polarization [9].
- **Th2 cells** are major players in immunity against parasites especially helminths and also contribute to allergic reactions. Th2 cells produce IL-4, IL-13 (stimulate B cells to produce IgE, persitaltism enhancement and mucous production) and IL-5 (eosinophil recruitment and development). Th2 specific transcription factor is **GATA3** [37]. STAT6 is an important factor for Th2 signaling, which inhibits Th1 polarization [9].
- **Th17 cells** eradicate extracellular bacteria (neutrophil enriched inflammations) and participate in antifungal responses. Th17 specific transcription factor is retinoic acid receptor-related orphan receptor γ (**RORγt)** [38]. STAT3 is required for the induction of RORγt. It Suggests that STAT3 and RORγt cooperate to induce IL-17 expression, which is a hallmark cytokine in Th17 cells [39].
- **Treg cells** regulate other T cell subsets and suppress potentially pathological immune responses. Indeed, their function is tolerance maintenance. Treg specific transcription factor is **FoxP3** [37]. STAT5 binds to the promoter of the *FoxP3* gene and it can promote Treg differentiation by regulating FoxP3 expression [40].

In the following, the role of this pathway in Th development is more explained.

Although JAK3 is activated upon cytokine induction such as IL-4, IL-2 and IL-7 and is frequently expressed in hematopoietic and immunologic cells, JAK2 is the most ubiquitous kinase activated by two thirds of ligands. JAK1 and Tyk2 can be found ubiquitously but they are limited in comparison to JAK2 [41]. Genome-wide association studies (GWAS) link TYK2 to several autoimmune and inflammatory disorders. It also plays important roles in the prevention and activation of carcinogenesis as well as unexpected tumor rejection [42]. TYK2, JAK2, and STAT4 are important for interleukin-12 (IL-12) signaling, resulted in Th1 cell differentiation, whereas JAK1, JAK3, and STAT6 are important for IL-4 signal transduction, resulted in Th2 cell differentiation [9]. However, it has been thought that Th2 polarization could be happened in the absence of STAT6 [43]. In fact, the role of STATs is complicated in Th regulation. IL-2 upregulates GATA3 and promotes the expression of IL-4 receptor thus IL-2 enhances Th2 polarization [44]. STAT5A and STAT5B are activated by IL-2 which facilitate binding to the promoter of Il4ra gene and make it to be overexpressed. In addition to above STAT3 can also induce Th2 polarization [45]. Therefore, despite the previous studies emphasizing only on STAT6 role in differentiation of Th2 cells, it is now clear that this polarization demands more interactions of STAT3 and STAT5A/B beside STAT6. Surprisingly, STAT5A and STAT5B are also involved in Th1 and Treg cells by promoting the expression of *T-bet* and *FoxP3* genes, respectively [6]. Whilst the latter is STAT5 dependent, T-bet is far more dependent on STAT1. RORc is more likely to be inhibited by STAT5 than induced by this factor, therefore, STAT5A and STAT5B are important negative regulators of Th17 differentiation [46]. Binding of type I IFNs to subunits of the receptor, IFNAR1 and IFNAR2, induces the activation of TYK2 and JAK1, respectively. These kinases phosphorylate STAT1 and STAT2. Type I IFNs can also activate STAT4, which implies that they might also regulate Th1 cell polarization [47]. However, their potency is low in comparison with IL-12, because of transient activation of STAT4 by type I IFNs (Fig. 3) [48]. The activity of Treg cells is inhibited by type I IFNs. Selective IFNAR deficiency in Treg cells was associated with defective antiviral function of T cells [49]. It was one of the myriad complicated signaling cascades, determining the fate of T helper cell polarization. However, exact regulators that modulate JAK-STAT pathway are still unknown.

### Inhibitors of the JAK-STAT pathway

#### Suppressors of Cytokine Signaling (SOCSs)

SOCS protein family are eight members, including SOCS1, SOCS2, SOCS3, SOCS4, SOCS5, SOCS6, SOCS7 and cytokine-inducible SH2 domain protein (CIS or CISH) [50, 51]. They play pivotal roles in immune regulation confirmed by genetical and biochemical studies. CIS binds to phosphorylated tyrosines located on the receptors and consequently, compete with STATs in binding to docking sites. It was previously shown that CIS deficient mice had no impaired function of the immune system [33]. However, recent studies have shown that CIS is an important regulator of both STAT-dependent and -independent immune responses [52, 53]. SOCS proteins contain an approximately 40 amino acid-box, named **SOCS box**, at C-terminus and a central **SH2 domain** flanked by a variable length sequence located at N-terminus (Fig. 1) [26, 54]. SOCS box is a unique motif which is not only found in this family, but also can be explored in other miscellaneous proteins [55]. SH2 domain directly binds to phosphorylated tyrosines of activated JAKs, therefore both recruitment of signal transducer adaptors such as STATs and kinase activity of JAKs is blocked [56, 57]. Additionally, SH2 determines the target of degradation [58]. SOCS box provides interactions leading to proteasomal degradation. In fact, this motif interacts with elongin B and C complex, Cullins, and the RING finger domain Box-2 (RBX2) protein (which recruits E2-ubiquitin transferase) and mediate JAK ubiquitination by E3-ubiqutin ligase activity (Fig. 2) [55, 59]. Altogether, SOCS proteins preferentially regulate the termination of JAK-STAT signaling process. On the other hands, SOCS deficiency or reduced SOCS expression may lead to changes in cytokine responses [33]. SOCS proteins are not expressed at high levels in unstimulated levels, however, their transcription is promptly induced upon cytokine stimulation and JAK-STAT activation [56, 57]. The role of SOCS proteins in the fate of T helper cells is very complicated (Fig. 3). SOCS1 directly binds to the SH2 domain of activated JAKs and therefore, the activation of JAK is inhibited [56, 57]. Studies on SOCS1^−/−^ Knockout mice revealed that IFN-γ and STAT1 are constitutively expressed in these KO mice. Hence, SOCS1 deficiency leads to Th1 differentiation and prevents Th17 differentiation. SOCS1 inhibits Th1 differentiation through inhibition of the IFN-γ–STAT1 and IL-12–STAT4 pathways [60, 61]. SOCS1 has a central role in Treg cell regulation. SOCS1 is named as a guardian of Treg cells, because SOCS1 prevents Tregs from producing of IFN-γ by suppression of STAT1. Deletion of SOCS1 enhances Treg proliferation, however, their function is defective [62]. *SOCS1* ^−/−^ Treg cells indicate over production of IFN-γ because of uncontrolled STAT1 activity. SOCS1 negatively regulate the number of Treg cells, but has an essential role in Treg functions [63]. SOCS3 either binds to the receptors directly or binds JAK1, JAK2, and TYK2 and inhibits them, but JAK3 is not affected [64]. Whenever JAKs are activated upon receptor stimulation, SOCS3 is precisely phosphorylated on Tyr204 and Tyr 221 residues located in SOCS box, thus, interaction with elongin B/C is promoted and the degradation of SOCS3 is accelerated [5, 56]. There was a significant correlation between the expression of SOCS3 and severity of human allergic reactions, such as atopic dermatitis and asthma. SOCS3 inhibits Th1 differentiation by suppression of STAT4 –which is mediated by IL-12 - and enhance Th2 skewing [65]. SOCS3 can also suppress IFN-γ signaling, but in comparison to SOCS1 is less potent [66]. Interestingly, SOCS3 inhibits Th17 differentiation by STAT3 suppression (53). The absence of SOCS3 promotes Th17 development, causing highly pro-inflammatory responses [62]. However, several studies showed that SOCS3 plays contradictory roles in T cell regulation [67]. This contradiction is due to dual effect of STAT3 which enhances the production of both anti-inflammatory cytokines (such as TGF-β and IL-10) and also inflammatory cytokines such as IL-17 and IL-6. SOCS3 is responsible for the regulation of this paradigm [58]. It seems that the strength and duration of STAT signaling is a key factor in balance of inflammatory and anti-inflammatory events [68]. For example, IL-6 and IL-10 are both the activator of STAT3. But, how IL-10 can activate anti-inflammatory genes and in contrast IL-6 can activate inflammatory genes? Therefore, it is postulated STAT3 activation by IL-10 varies with STAT3 activation by IL-6 [41]. One logical answer to this question may be that IL-6 signaling is regulated by SOCS3, whereas IL-10 signaling is not affected [58].

The role of SOCS3 in the development and function of Treg cells is still undetermined [69]. SOCS3 may suppress Treg proliferation. It also suppresses CTLA4 and FoxP3 levels by repressing IL-2 expression [70]. SOCS3 inhibits IL-6 signaling. Therefore, the dysfunction of SOCS3 may result in Th17/Treg imbalances because of alterations in reciprocal IL-6/STAT3 and IL-2/STAT5 pathways [71]. SOCS4 is the least studied protein, which has been cleared to be important for Epidermal Growth Factor Receptor (EGFR(signaling. Phosphorylated tyrosines on EGFR are docking sites for SOCS4 binding, subsequently, SOCS4 causes proteasomal degradation of EGFR by recruitment of E3 ubiquitin ligases [72]. SOCS5 has a crucial role in Th1 skewing responses. SOCS5 is induced by IL-6 signaling and negatively regulates STAT6 which is essential for IL-4 signaling and therefore inhibits Th2 differentiation [73, 74]. Furthermore, SOCS5 is required for EGFR degradation. It binds to both EGFR and elongin B/C complex. This complex may facilitate the rapid degradation of SOCS5 protein itself. It seems that other SOCS proteins undergo degradation by elongin B/C complex to ensure their short life span [72]. The last SOCS family member is CIS protein. CIS is induced by IL-4 and negatively regulates the activation of STAT3, STAT5 and STAT6 in T cells. They showed that CIS-deficient mice spontaneously developed airway inflammation and also CIS deficiency in T cells led to greater susceptibility to experimental allergic asthma. The lack of CIS in T cells causes more TH2 differentiation and exacerbated airway allergic diseases. Therefore, CIS has a critical role in controlling the proallergic generation of helper T cells [52]. Cis physically interacts with PLC-γ1, an intermediate adaptor for TCR signaling, targeting it for proteasomal degradation after TCR stimulation. Genetic deletion of Cis in CD8+ T cells augments their expansion, function and cytokine responsiveness, resulting in significant regression of established tumors. Therefore, it is a suitable candidate for adoptive cancer immunotherapy [53]. Altogether, SOCS proteins possess important roles in modulating immune responses, especially the fate of Th cell polarization. Their function is specific for cytokines but not for JAKs/STATs.

### Protein Inhibitors of Activated STAT (PIASs)

The mammalian PIAS protein family contains 4 members, including PIAS1 (Gu binding Protein) [75], PIAS2 (PIASx),PIAS3, and PIAS4 (PIASy) [76]. Unlike SOCS family members, PIAS proteins are constitutively expressed [77]. They possess several domains, including a serine/threonine rich domain located at C-terminus which is responsible for target binding, at the central portion there is a Zn-binding RING-finger like domain (RLD) which is responsible for SUMO transfer and near the N-terminus, there is conserved SAP domain (**S**caffold attachment factor A/B, **A**cinus, **P**IAS) [78] which is an important part for target binding via scaffold/matrix attachment regions (S/MARs). These regions are located on chromatin and bind to the nuclear matrix [79]. The PIAS proteins bind to dimers of activated STATs (not monomer STAT) and prohibit them from binding to specific sequences in DNA. However, the exact mechanism by which PIAS proteins do their regulative functions remains unrevealed [8]. In vivo co-immunoprecipitation investigations introduced specific PIAS–STAT interactions in mammalian cells. Upon cytokine signaling, PIAS1, PIAS3, PIASX and PIASy interact with STAT1, STAT3, STAT4 and STAT1, respectively [18, 80–82].

Although it has been claimed that PIAS family members inhibit gene expression by a STAT-dependent manner, discrete mechanisms are complicated. For example, PIAS1 and PIAS3 inhibit the DNA binding capability of STAT1 and STAT3, respectively [18, 82] while PIASX and PIASY inhibit STAT4- and STAT1-dependent gene transcription without alteration the DNA binding capability of STAT4 and STAT1 [81, 82]. In addition to the ability of PIAS proteins in STAT blocking, they have SUMO E3 ligase activity contributing to a phenomenon named SUMOylation, which is discussed later [83, 84]. A number of studies showed the interaction of PIAS proteins with special histone deacetylases (HDACs) (Fig. 2). They possibly act as a transcriptional corepressor of STATs [33]. The interactions of HDAC3 with PIASx [85] and also HDAC1with PIASy have been described [86]. Nonetheless, further bimolecular studies are needed to survey the physiological function of mammalian PIAS proteins in the regulation of STAT activities.

### Protein tyrosine phosphatases (PTPs)

PTPs are the third protein groups which negatively regulate the JAK-STAT pathway functions. They are comprised of several distinct proteins and dephosphorylate tyrosine residues involved in signaling pathways, consequently reverse JAK-STAT activity [87]. Some of the PTPs are CD45, SHP1and SHP2 (Fig. 2) [88]. CD45 is a receptor which is highly expressed by hematopoietic cells, especially B and T cells [89]. It directly binds to all kinds of JAKs and also can remove the phosphorus from the regulator tyrosine of Src family, which facilitates their reactivation in the next signaling trigger. The family of Src tyrosine kinase participates in the regulation a large number of signaling pathways in the immune cells [90]. Studies on CD45^−/−^ cells revealed that JAK phosphorylation is substantially increased and CD45 ablation enhances the generation of erythroid colonies and also antiviral potency which emphasizes on the role of CD45 in the negative regulation of EPO and IFN signaling, respectively [33]. However, the role of CD45 in the regulation of cytokine signaling needs further clarification.

SHP1 and SHP2 are distinguished by tandem SH2 domains located at the N-terminus which enable them to bind specific signaling proteins, especially cytokine receptors; a PTP domain which is catalytic region and a C-terminal region containing several tyrosine residues among divergent sequences (Fig. 1). When the latter region is phosphorylated, it acquires a key role as a docking site for interaction with other signaling molecules [87, 91, 92]. For example, SHP1 that is expressed in hematopoietic lineage contains two SH2 domains and not only binds to phosphorylated JAKs, but also dephosphorylates activated signaling proteins. The best model used to confirm SHP1 deficiency is motheaten mouse [8]. SHP1 is physically associated with the other receptors, including c-KIT-R, β chain of IL-3R and also the erythropoietin receptor (EPOR) [93]. Genetic studies revealed that JAK1 is negatively regulated by SHP2. The level of phosphorylated JAK1 is increased in *SHP2* ^−/−^ fibroblasts after IFN-γ stimulation [94]. **SH2B** [PSM (proline-rich, PH and SH2 domain-containing signaling mediator) or SH2B1] /**Lnk** (SH2B3)/**APS** (Adaptor protein with PH and SH2 domain) are also SH2 containing proteins which are involved in the JAK-STAT pathway regulation (Fig. 2). In addition to SH2 domain (near to C-terminal) they contain pleckstrin homology domain and dimerization domain (at N-terminus) and serve as a substrate for JAKs to be phosphorylated [95]. Pleckstrin domain facilitates phospholipid binding [96]. There are several isoforms of SH2B but it has been reported that SH2-Bβ positively regulates JAK2. Additionally, other studies suggest that Lnk and APS are negative regulators of this pathway and other signaling pathways. Lnk and APS regulate the proliferation, survival and migration of B cell progenitors (IL-7/SCF signaling pathways) and mature (BCR signaling) cells [97]. The role of Lnk has been established in autoimmune diseases, however, the function of other isoforms of SH2B family is yet to be defined [8].

There are other proteins modulating the function of signaling molecules. Protein-tyrosine phosphatase 1B (PTP1B) and T-cell PTP (TCPTP) were reported through gene targeting studies. In fact, they are categorized in PTP group and suggested to be important in the JAK dephosphorylation process. PTP1B can negatively affect the regulation of JAK2 and TYK2 but JAK1 is not implicated [98]. *Ptp1b* ^−/−^ mice showed enhanced phosphorylation of JAK2 in their Embryo fibroblasts. For instance, Leptin signaling may be regulated by this mechanism [99]. Although any immunological phenotype of PTP1B has not been observed, TCPTP has been found in macrophages. TCPTP can dephosphorylate JAK1 and JAK3 [93]. TCPTP deficiency causes impaired T- and B-cell functions and hematopoietic defects [100]. In addition to above, newly supposed mechanisms are involved in the JAK-STAT regulation. Some of them are discussed in the following.

**Ubiquitination**: Ubiquitin is a highly conserved polypeptide (76 amino acids and 8.5 kDa) which is covalently conjugated to the residues of protein substrates [101, 102]. This ubiquitin dependent proteolysis has fundamental roles in cellular activities, including antigen presentation, expression of surface receptors, transcription and especially signaling pathways. The process of ubiquitination is ATP dependent and needs sequential functions of three enzymes, including activating enzyme (E1), conjugating enzyme (E2) and ligase (E3). Ubiquitin itself can be ubiquitinated so as to form ubiquitin chains attached to targeted proteins and these oligo- or poly-ubiquitin residues can be further subdivided into homotypic (single type of ubiquitination linkage) or heterotypic (mixed type of ubiquitination linkage) chains. For polyubiquitination, the type of linkage determines the consequence of protein labeling. Whenever C-terminal glycine of a ubiquitin molecule binds covalently to one of seven lysine residues of another ubiquitin molecule, polyubiquitination is established. Therefore, seven different types of ubiquitination may be occurring: K6-, K11-, K27-, K29-, K33-, K48-, and K63 ubiquitination. The most well-defined linkage type is K48 ubiquitination and it prepares proteins to be degraded [103, 104]. Then, proteins are transferred to an enzymatic complex known as the 26S proteasome. At the end of the process ubiquitins remain intact and are recycled (Fig. 2). SOCS are attached to a ubiquitin E3 ligase to target signaling proteins to be degraded [33]. For example, the expression of SOCS1enhances the degradation of ubiquitinated JAK2 [105]. K63 ubiquitination mediates various cellular processes, including innate immunity, autophagy, endocytosis and also DNA repair. The function of other types is less understood [103]. Mono-ubiquitination is another form of ubiquiltylation which doesn’t lead to protein degradation, but rather can modulate protein localization such as TCR endocytosis [106].

**ISGylation (Interferon Stimulated Gene 15):** ISG15 is a 17,145 Da ubiquitin like eukaryotic protein, which is induced by type I Interferons, viral infections and lipopolysaccharide encounter [107] and it can modify the function and localization of its target proteins after covalent attachment [108]. The biological activities of ISG15 are poorly described [109] but it is supposed that the function of ISGylation is similar to monoubiquitination [110] and engages enzymatic cascade, including E1, E2 and E3 enzymes facilitating ISG15 conjugation to lysine residues on target proteins [106]. Several studies reported some of ISGylated proteins, such as JAK1 and STAT1 [111]. Neither the precise ISGylation site (s) located on JAK1 or STAT1, nor the activities of JAK1 or STAT1 by which are regulated upon ISGylation are fully defined. Nevertheless, ISGylation appears to serve as a positive-feedback mechanism in the regulation of the JAK–STAT pathway [33]. Furthermore, ISG12a is another ISG family member that promotes the production of IFN α/β and activates the type I IFN signaling pathway as shown by increasing p-STAT1 levels, up-regulating ISG levels and elevating Interferon sensitive response element (ISRE) activity. ISG12a can inhibit the replication of HCV and promote the anti-HCV activity of IFN-α probably by the production of type I IFNs and activation of Jak/STAT signaling pathway [112].

**SUMOylation (Small Ubiquitin-related Modifier):** SUMO is a ubiquitin-related molecule which is conjugated to proteins in a manner known as SUMOylation [113]. The exact mechanism of SUMOylation is not well defined, but has been suggested to cause various functions, such as transcription regulation, targeting of nuclear proteins, and regulation of protein-protein interactions, etc. STAT1 is inhibited by SUMO conjugation. These findings introduce SUMO conjugation of STAT1 as a mechanism to constitutively attenuate the IFNγ sensitivity of cells. In fact, it prevents hyperresponsiveness to IFNγ [114]. Although it is not clear whether SUMOylation plays a role in the development of the immune system, a study showed that SUMO2 can modify STAT5, resulting in a block in its acetylation and subsequent signaling of the early T and B cells [115]. Additionally, PIAS proteins seem to have SUMO E3 ligase activity [116]. For example, it was shown that STAT1 is SUMOylated by PIAS under treatment with IFN-γ [95]. Whether SUMOylation has an effective role in the regulation of the JAK-STAT pathway has remained to be more clear.

### Therapeutic approaches

GWAS studies suggested that most of cytokine receptors play their important roles in the initiation and development of various immunological diseases through the JAK-STAT pathway, especially T cell mediated diseases. Therefore, targeting of this pathway has gained huge attraction [117]. Monoclonal antibodies are effective in the regulation of cytokines and their receptors extracellularly but intracellular signaling proteins can also be targeted in the clinical settings. For this purpose, new drugs were introduced to inhibit JAK and STAT molecules (Table 1). Although only few JAK inhibitors (JAKinib) are FDA approved, other JAKinibs and possible STAT inhibitors are being developed by passing preclinical evaluations and clinical trials [118].

Ruxolitinib is the first FDA approved JAK inhibitor that inhibits JAK1 and JAK2. It was approved for the treatment of intermediate- and high-risk primary myelofibrosis and it has also been studied in RA and psoriasis yielding hopeful results. It is effective in the treatment of JAK2-gain of function mutations such as myelofibrosis, Polycythemia vera and in topical application for treatment of psoriasis [117, 119]. Ruxolitinib is an effective drug, with a better outcome in myelofibrosis patients with a low-intermediate1 risk score; thus, early administration of Ruxolitinib may be worthy in this course of myelofibrosis [120]. However, another study acclaims that it is useful in the treatment of patients with intermediate or high risk myelofibrosis [121]. Baricitinib is a JAK1/JAK2 inhibitor, which has shown encouraging results in the treatment of RA [122]. Phase III and II trials are also ongoing for RA, psoriasis and in the treatment of diabetic nephropathy, SLE and atopic dermatitis [123].

Tofacitinib is a selective JAKinib and it was tested and approved for human disease treatment. Tofacitinib is widely studied and is effective for controlling both innate and adaptive immunity. JAK3 is affected more than JAK1 and JAK2 is the least affected JAK by Tofacitinib [124, 125]. Tofacitinib ameliorates murine lupus and improves vascular dysfunction. It also regulates the formation of Neutrophil Extracellular Traps (NETs) and significantly improves endothelial differentiation and endothelium-dependent vasorelaxation. Therefore, it may be beneficial in SLE and its associated vascular dysfunction [126]. Since JAK3 unlike the other JAKs is limited to cytokines of the common γ chain family; therefore, Tofacitinib inhibits IL-2, IL-4. IL-7, IL-9, IL-15 and IL-21 cytokines, which use common γ chain receptor. Furthermore, IFN-γ, IL-6, IL-13 and to a lesser extent IL-12 and IL-23 can be blocked [127]. An interesting property of Tofacitinib is no effect on other kinases except JAKs. Tofacitinib is the first JAKinib which was approved for the treatment of rheumatoid arthritis (RA) [123]. In this way, combined molecular modeling techniques such as 3D-QSAR, molecular docking and molecular dynamics help us to design more potent JAKinibs [128].

Oclacitinib was recently approved as a pan-JAK inhibitor for the treatment of atopic dermatitis in canines. It is not currently being used in humans, however its effectiveness in treating allergic skin diseases has made it a potent candidate to be used in immune mediated dermatologic disorders [129]. In a recent study of treatment for canine atopic dermatitis, the efficacy of oclacitinib was compared to ciclosporin which oclacitinib showed a faster onset of action and a lower frequency of gastrointestinal side effects [130]. Momelotinib is a JAK1/2 inhibitor that surprisingly showed a reduction of anemia in MF patients during Phase I/II trials. Current phase III trials are in progress for approval of momelotinib during 2017 [131]. Peficitinib can inhibit JAK1 and JAK3, STAT5 phosphorylation in vitro and ex vivo and IL-2-dependent T cell proliferation in vitro. Peficitinib has therapeutic potential for the treatment of RA [121]. Some of JAKinibs and potent STAT inhibitors are listed in Table 1. Using of JAKinibs may be associated with some degree of adverse reactions. The most common adverse reaction is susceptibility to infections but opportunistic infections may also occur [6].

**Table 1:** A number of Jakinibs and STAT inhibitors

Drug	Targt	Disease	Status
Ruxolitinib (INC424)	JAK1, JAK2	Polycythemia, Psoriasis (topical), myelofibrosis, Various cancers,	*FDA approved*
Tofacitinib	JAK3 *>* JAK1> > (JAK2)	RA, Psoriasis, Spondyloarthropathy, Transplant rejection, ulcerative colitis	*FDA approved*
Oclacitinib	JAK1	Canine allergic dermatitis	*FDA approved*
Baricitinib	JAK1, JAK2	RA Psoriasis, diabetic nephropathy, SLE, Atopic dermatitis	Phase III Phase II
Momelitinib	JAK1, JAK2	Myelofibrosis	Phase III
Peficitinib	JAK1, JAK3	RA Psoriasis	Phase III Phase II
INCB039110	JAK1, JAK2	Psoriasis, RA	Phase II
AZD1480	JAK1, JAK2	Myeloproliferative diseases, various cancers	Phase I
ISIS-STAT3Rx (AZD9150)	STAT3	Various cancers	Phase II
OPB-31121	STAT3	Various cancers	Phase I

Although several STAT inhibitors have been introduced to date [132], no STAT inhibitor has been developed successfully [118]. Limitation in such a success is due to issues with selectivity, bioavailability, similar homology and in vivo efficacy. For example, STAT3 has strict homology to STAT1; Since STAT1 takes part in Interferon signaling; therefore, inappropriate targeting of STAT3 may result in dysregulation in normal cellular processes such as apoptosis and anti-microbial defense. Although STAT3 is an intriguing therapeutic target for cancer therapy, none of its inhibitors are clinically successful because of low potency or poor druggability. Herein, a series of aminobenzo[b]thiophene 1, 1-dioxides with good drug-likeness properties were designed, synthesized and evaluated as STAT3 inhibitors. Most of them exhibited higher antitumor activity without influencing the phosphorylation levels of the upstream kinases Src and Jak2 [133]. Likewise, there is another JAK2-STAT3 inhibitor named dihydroartemisinin. This putative STAT3 inhibitor may be a new and effective drug for cancer treatment and a therapeutic approach in head and neck squamous cell carcinoma (HNSCC) [134]. STA-21 is a dynamic STAT-3 inhibitor, which influences the expansion and progression of RA. Interestingly, STA-21 is useful either in the regulation of Th1/Th2 serum cytokine levels or the mRNA and protein expression of critical factors, including RORγt, T-bet, IL-4, GATA-3, JAK1 and STAT3. It can inhibit the production of TNF-α and IL-6 in the peripheral blood of Balb/c mice. Administration STA-21 prevents cellular signaling pathways and downstream activation of specific Th transcription factors previously indicated to play key roles in the pathogenesis of RA. Therefore, STA-21 could be administered as a potential treatment for RA patients [135]. STAT-5 deletion in white adipose tissue may increase adiposity, whereas decrease insulin resistance and gluconeogenic capacity. In the light of STAT5 role in maintaining lipid homeostasis in white adipose tissue, STAT5 manipulation may improve the outcomes in metabolic diseases [136]. BJ-3105, a 6-alkoxypyridin-3-ol analog, inhibited the production of IFN-γ and IL-17 from polyclonal CD4^+^ Th cells and improved the experimental autoimmune encephalomyelitis (EAE) model by reducing Th1 and Th17 generation, respectively. In fact, BJ-3105 inhibited the phosphorylation of JAK and its downstream STAT that play pivotal roles in Th differentiation [137]. Finally, we can also use other derivatives to shift the pathogenic Th1/Th17 responses to Th2/Treg responses by affecting the JAK-STAT pathway [138].

Although STAT targeting is complicated, it is achievable by the following strategies: 1st phosphorylation blocking, 2nd disrupting of SH2 domain binding to phosphorylated residues on activated receptors and therefore blocking of dimrerization, 3rd interference in DNA binding capability of STATs [132, 139]. Regarding to the pathophysiologic roles of other regulators involved in JAK-STAT pathway, there is a high potential to do more research and experiments in order to facilitate precise targeting of this pathway.

## Conclusions

JAK-STAT pathway has critical roles in the regulation of immune system, especially the fate of T helper cells. Th cells play a central role in direction of the immune responses. JAKs are associated with cytokine receptors, which are activated upon stimulation and they phosphorylate STAT proteins, enabling them to be transported to the nucleus. Several regulators, such as PTPs, SOCS and PIAS families have been described to modulate the function of the JAK-STAT pathway. Since any dysregulation in the JAK-STAT pathway and their regulators may lead to pathological consequences; therefore, signaling pathways are potential therapeutic approaches which targeting of them may lead to develop new strategies in the treatment of different diseases, particularly T cell mediated diseases. Targeting strategies may result in numerous benefits, for instance side effect and unwanted reactions may be diminished and bystander pathways remain intact. We hope that the severity and burden of the diseases can be alleviated and decreased by the development of new drugs and precise targeting of these proteins in pathological circumstances. Studies and experiments on the regulation of the JAK-STAT pathway are ongoing and other aspects of their functions need further elucidation.

## Abbreviations

APS: Adaptor protein with PH and SH2 domain
CIS: Cytokine-inducible SH2 domain protein
EAE: Experimental Autoimmune Encephalomyelitis
EGFR: Epidermal Growth Factor Receptor
HDACs: Histone deacetylases
HNSCC: Head and Neck Squamous Cell Carcinoma
ISGylation: Interferon Stimulated Gene 15
JAK: Janus kinase
NET: Neutrophil Extracellular Traps
PIAS: Protein Inhibitors of Activated STATs
PTK: Protein Tyrosine Kinases
PTP1B: Protein tyrosine phosphatase 1B
PTPs: Protein tyrosine phosphatases
RORγt: Retinoic acid receptor-related Orphan Receptor γ
SAP domain: Scaffold attachment factor A/B, Acinus, PIAS
SH2 domain: Src homology 2 domain
SH2B: Proline-rich, PH and SH2 domain-containing signaling mediator
SOCS: Suppressors of Cytokine Signaling
STAT: Signal Transducer and Activator of Transcription
StIP: STAT Interacting Protein
SUMO: Small Ubiquitin-related Modifier
TAD: Trans-Activation Domain
TCPTP: T-cell Protein Tyrosine Phosphatase
Th cell: T helper cell

## References

[1] Renauld J-C (2003). Class II cytokine receptors and their ligands: key antiviral and inflammatory modulators. Nat Rev Immunol.

[2] O'Shea JJ, Gadina M, Schreiber RD (2002). Cytokine signaling in 2002: new surprises in the Jak/Stat pathway. Cell.

[3] Ghoreschi K, Laurence A, O’Shea JJ (2009). Janus kinases in immune cell signaling. Immunol Rev.

[4] Liongue C, O'Sullivan LA, Trengove MC, Ward AC (2012). Evolution of JAK-STAT pathway components: mechanisms and role in immune system development. PLoS One.

[5] Sasaki A, Yasukawa H, Shouda T, Kitamura T, Dikic I, Yoshimura A (2000). CIS3/SOCS-3 suppresses erythropoietin (EPO) signaling by binding the EPO receptor and JAK2. J Biol Chem.

[6] O'Shea JJ, Plenge R (2012). JAK and STAT signaling molecules in immunoregulation and immune-mediated disease. Immunity.

[7] Schwartz DM, Bonelli M, Gadina M, O'shea JJ (2016). Type I/II cytokines, JAKs, and new strategies for treating autoimmune diseases. Nat Rev Rheumatol.

[8] Rawlings JS, Rosler KM, Harrison DA (2004). The JAK/STAT signaling pathway. J Cell Sci.

[9] O'Shea JJ, Murray PJ (2008). Cytokine signaling modules in inflammatory responses. Immunity.

[10] Levy DE, Kessler DS, Pine R, Reich N, Darnell J (1988). Interferon-induced nuclear factors that bind a shared promoter element correlate with positive and negative transcriptional control. Genes Dev.

[11] Edmonson J, LoTurco J, Blanton M, Kriegestein A, Moran D (1991). Interferon-dependent tyrosine phosphorylation of latent cytoplasmic transcription factor. Science.

[12] Stark GR, Darnell JE (2012). The JAK-STAT pathway at twenty. Immunity.

[13] Levy DE, Darnell J (2002). Stats: transcriptional control and biological impact. Nat Rev Mol Cell Biol.

[14] Nardozzi JD, Lott K, Cingolani G (2010). Phosphorylation meets nuclear import: a review. Cell Communication and Signaling.

[15] O'shea JJ, Holland SM, Staudt LM (2013). JAKs and STATs in immunity, immunodeficiency, and cancer. N Engl J Med.

[16] Zhu X, Wen Z, Xu LZ, Darnell J (1997). Stat1 serine phosphorylation occurs independently of tyrosine phosphorylation and requires an activated Jak2 kinase. Mol Cell Biol.

[17] Decker T, Kovarik P. Serine phosphorylation of STATs. Oncogene. 2000;2628–37. 10.1038/sj.onc.1203481 10851062

[18] Chung CD, Liao J, Liu B, Rao X, Jay P, Berta P (1997). Specific inhibition of Stat3 signal transduction by PIAS3. Science.

[19] Dudley AC, Thomas D, Best J, Jenkins A (2004). The STATs in cell stress-type responses. Cell Communication and Signaling.

[20] Stark GR, Kerr IM, Williams BR, Silverman RH, Schreiber RD (1998). How cells respond to interferons. Annu Rev Biochem.

[21] Pearson MA, Reczek D, Bretscher A, Karplus PA (2000). Structure of the ERM protein moesin reveals the FERM domain fold masked by an extended actin binding tail domain. Cell.

[22] Tepass U (2009). FERM proteins in animal morphogenesis. Curr Opin Genet Dev.

[23] Liu BA, Jablonowski K, Raina M, Arcé M, Pawson T, Nash PD (2006). The human and mouse complement of SH2 domain proteins—establishing the boundaries of phosphotyrosine signaling. Mol Cell.

[24] Shuai K, Horvath CM, Huang LHT, Qureshi SA, Cowburn D, Darnell JE (1994). Interferon activation of the transcription factor Stat91 involves dimerization through SH2-phosphotyrosyl peptide interactions. Cell.

[25] Kisseleva T, Bhattacharya S, Braunstein J, Schindler C (2002). Signaling through the JAK/STAT pathway, recent advances and future challenges. Gene.

[26] Lim WA, Pawson T (2010). Phosphotyrosine signaling: evolving a new cellular communication system. Cell.

[27] Darnell JE (1997). STATs and gene regulation. Science.

[28] Shuai K, Stark GR, Kerr IM, Darnell JE (1993). A single phosphotyrosine residue of Stat91 required for gene activation by interferon. Science.

[29] Xu X, Sun Y-L, Hoey T (1996). Cooperative DNA binding and sequence-selective recognition conferred by the STAT amino-terminal domain. Science.

[30] Shuai K, Liao J, Song MM (1996). Enhancement of antiproliferative activity of gamma interferon by the specific inhibition of tyrosine dephosphorylation of Stat1. Mol Cell Biol.

[31] Horvath CM (2000). STAT proteins and transcriptional responses to extracellular signals. Trends Biochem Sci.

[32] Horvath CM, Wen Z, Darnell J (1995). A STAT protein domain that determines DNA sequence recognition suggests a novel DNA-binding domain. Genes Dev.

[33] Shuai K, Liu B (2003). Regulation of JAK–STAT signalling in the immune system. Nat Rev Immunol.

[34] Li WX (2008). Canonical and non-canonical JAK–STAT signaling. Trends Cell Biol.

[35] Boyton RJ, Altmann DM (2002). Is selection for TCR affinity a factor in cytokine polarization?. Trends Immunol.

[36] Mullen AC, High FA, Hutchins AS, Lee HW, Villarino AV, Livingston DM (2001). Role of T-bet in commitment of TH1 cells before IL-12-dependent selection. Science.

[37] Zhou L, Chong MM, Littman DR (2009). Plasticity of CD4+ T cell lineage differentiation. Immunity.

[38] Chen Z, Laurence A, O'Shea JJ. Signal transduction pathways and transcriptional regulation in the control of Th17 differentiation. Seminars Immunol. 2007;19:400–408. 10.1016/j.smim.2007.10.015 PMC2323678 18166487

[39] Bell E (2007). New player in the generation of TH17 cells. Nat Rev Immunol.

[40] Burchill MA, Yang J, Vogtenhuber C, Blazar BR, Farrar MA (2007). IL-2 receptor β-dependent STAT5 activation is required for the development of Foxp3+ regulatory T cells. J Immunol.

[41] Herrington J, Smit LS, Schwartz J, Carter-Su C (2000). The role of STAT proteins in growth hormone signaling. Oncogene.

[42] Leitner NR, Witalisz-Siepracka A, Strobl B, Müller M. Tyrosine kinase 2–Surveillant of tumours and bona fide oncogene. Cytokine. 2015;89:209–218. 10.1016/j.cyto.2015.10.015 26631911

[43] van Panhuys N, Tang S-C, Prout M, Camberis M, Scarlett D, Roberts J (2008). In vivo studies fail to reveal a role for IL-4 or STAT6 signaling in Th2 lymphocyte differentiation. Proc Natl Acad Sci.

[44] Paul WE (2010). What determines Th2 differentiation, in vitro and in vivo&quest. Immunol Cell Biol.

[45] Liao W, Schones DE, Oh J, Cui Y, Cui K, Roh T-Y (2008). Priming for T helper type 2 differentiation by interleukin 2–mediated induction of interleukin 4 receptor α-chain expression. Nat Immunol.

[46] Laurence A, Tato CM, Davidson TS, Kanno Y, Chen Z, Yao Z (2007). Interleukin-2 signaling via STAT5 constrains T helper 17 cell generation. Immunity.

[47] Crouse J, Kalinke U, Oxenius A (2015). Regulation of antiviral T cell responses by type I interferons. Nat Rev Immunol.

[48] Ramos HJ, Davis AM, George TC, Farrar JD (2007). IFN-α is not sufficient to drive Th1 development due to lack of stable T-bet expression. J Immunol.

[49] Srivastava S, Koch MA, Pepper M, Campbell DJ. Type I interferons directly inhibit regulatory T cells to allow optimal antiviral T cell responses during acute LCMV infection. Journal of Experimental Medicine*.*2014. doi:10.1084/jem.20131556. 10.1084/jem.20131556 PMC4010906 24711580

[50] Alexander WS (2002). Suppressors of cytokine signalling (SOCS) in the immune system. Nat Rev Immunol.

[51] Hilton D (1999). Negative regulators of cytokine signal transduction. Cell Mol Life Sciences CMLS.

[52] Yang XO, Zhang H, Kim B-S, Niu X, Peng J, Chen Y (2013). The signaling suppressor CIS controls proallergic T cell development and allergic airway inflammation. Nat Immunol.

[53] Palmer DC, Guittard GC, Franco Z, Crompton JG, Eil RL, Patel SJ (2015). Cish actively silences TCR signaling in CD8+ T cells to maintain tumor tolerance. J Exp Med.

[54] Kile BT, Schulman BA, Alexander WS, Nicola NA, Martin HM, Hilton DJ (2002). The SOCS box: a tale of destruction and degradation. Trends Biochem Sci.

[55] Kamizono S, Hanada T, Yasukawa H, Minoguchi S, Kato R, Minoguchi M (2001). The SOCS box of SOCS-1 accelerates ubiquitin-dependent proteolysis of TEL-JAK2. J Biol Chem.

[56] Starr R, Willson TA, Viney EM, Murray L, Rayner JR, Jenkins BJ (1997). A family of cytokine-inducible inhibitors of signalling. Nature.

[57] Endo TA, Masuhara M, Yokouchi M, Suzuki R, Sakamoto H, Mitsui K (1997). A new protein containing an SH2 domain that inhibits JAK kinases. Nature.

[58] Yasukawa H, Misawa H, Sakamoto H, Masuhara M, Sasaki A, Wakioka T (1999). The JAK-binding protein JAB inhibits Janus tyrosine kinase activity through binding in the activation loop. EMBO J.

[59] Kamura T, Sato S, Haque D, Liu L, Kaelin WG, Conaway RC (1998). The Elongin BC complex interacts with the conserved SOCS-box motif present in members of the SOCS, ras, WD-40 repeat, and ankyrin repeat families. Genes Dev.

[60] Tanaka K, Ichiyama K, Hashimoto M, Yoshida H, Takimoto T, Takaesu G (2008). Loss of suppressor of cytokine signaling 1 in helper T cells leads to defective Th17 differentiation by enhancing antagonistic effects of IFN-γ on STAT3 and Smads. J Immunol.

[61] Eyles JL, Metcalf D, Grusby MJ, Hilton DJ, Starr R (2002). Negative regulation of interleukin-12 signaling by suppressor of cytokine signaling-1. J Biol Chem.

[62] Tamiya T, Kashiwagi I, Takahashi R, Yasukawa H, Yoshimura A (2011). Suppressors of cytokine signaling (SOCS) proteins and JAK/STAT pathways regulation of T-cell inflammation by SOCS1 and SOCS3. Arterioscler Thromb Vasc Biol.

[63] Lu L-F, Thai T-H, Calado DP, Chaudhry A, Kubo M, Tanaka K (2009). Foxp3-dependent microRNA155 confers competitive fitness to regulatory T cells by targeting SOCS1 protein. Immunity.

[64] Babon JJ, Kershaw NJ, Murphy JM, Varghese LN, Laktyushin A, Young SN (2012). Suppression of cytokine signaling by SOCS3: characterization of the mode of inhibition and the basis of its specificity. Immunity.

[65] Seki Y-i, Inoue H, Nagata N, Hayashi K, Fukuyama S, Matsumoto K (2003). SOCS-3 regulates onset and maintenance of TH2-mediated allergic responses. Nat Med.

[66] Federici M, Giustizieri ML, Scarponi C, Girolomoni G, Albanesi C (2002). Impaired IFN-γ-dependent inflammatory responses in human keratinocytes overexpressing the suppressor of cytokine signaling 1. J Immunol.

[67] Kinjyo I, Inoue H, Hamano S, Fukuyama S, Yoshimura T, Koga K (2006). Loss of SOCS3 in T helper cells resulted in reduced immune responses and hyperproduction of interleukin 10 and transforming growth factor–β1. J Exp Med.

[68] El Kasmi KC, Holst J, Coffre M, Mielke L, de Pauw A, Lhocine N (2006). General nature of the STAT3-activated anti-inflammatory response. J Immunol.

[69] Lan F, Zhang N, Zhang J, Krysko O, Zhang Q, Xian J, et al. "Forkhead box protein 3 in human nasal polyp regulatory T cells is regulated by the protein suppressor of cytokine signaling 3," J Allergy Clin Immunol*,* vol. 132, pp. 1314-1321. e3, 2013. 10.1016/j.jaci.2013.06.010 23910692

[70] Pillemer BB, Xu H, Oriss TB, Qi Z, Ray A (2007). Deficient SOCS3 expression in CD4+ CD25+ FoxP3+ regulatory T cells and SOCS3-mediated suppression of Treg function. Eur J Immunol.

[71] Wang X-Q, Hu G-h, Kou W, Shen Y, Kang H-Y, Hong S-L (2012). Reciprocal roles of STAT3 and STAT5 in nasal polyposis. Am J Otolaryngol.

[72] Valdez BC, Henning D, Perlaky L, Busch RK, Busch H (1997). Cloning and characterization of Gu/RH-II binding protein. Biochem Biophys Res Commun.

[73] Nicholson SE, Willson TA, Farley A, Starr R, Zhang JG, Baca M (1999). Mutational analyses of the SOCS proteins suggest a dual domain requirement but distinct mechanisms for inhibition of LIF and IL-6 signal transduction. EMBO J.

[74] Kario E, Marmor MD, Adamsky K, Citri A, Amit I, Amariglio N (2005). Suppressors of cytokine signaling 4 and 5 regulate epidermal growth factor receptor signaling. J Biol Chem.

[75] Shuai K. Modulation of STAT signaling by STAT-interacting proteins. Oncogene. 2000;2638–44. 10.1038/sj.onc.1203522 10851063

[76] Lao M, Shi M, Zou Y, Huang M, Ye Y, Qiu Q (2016). Protein inhibitor of activated STAT3 regulates migration, invasion, and activation of fibroblast-like synoviocytes in rheumatoid arthritis. J Immunol.

[77] Kotaja N, Karvonen U, Jänne OA, Palvimo JJ (2002). PIAS proteins modulate transcription factors by functioning as SUMO-1 ligases. Mol Cell Biol.

[78] Kipp M, Göhring F, Ostendorp T, van Drunen CM, van Driel R, Przybylski M (2000). SAF-box, a conserved protein domain that specifically recognizes scaffold attachment region DNA. Mol Cell Biol.

[79] Liu B, Liao J, Rao X, Kushner SA, Chung CD, Chang DD (1998). Inhibition of Stat1-mediated gene activation by PIAS1. Proc Natl Acad Sci.

[80] Arora T, Liu B, He H, Kim J, Murphy TL, Murphy KM (2003). PIASx is a transcriptional co-repressor of signal transducer and activator of transcription 4. J Biol Chem.

[81] Liu B, Gross M, Ten Hoeve J, Shuai K (2001). A transcriptional corepressor of Stat1 with an essential LXXLL signature motif. Proc Natl Acad Sci.

[82] Tussié-Luna MI, Bayarsaihan D, Seto E, Ruddle FH, Roy AL (2002). Physical and functional interactions of histone deacetylase 3 with TFII-I family proteins and PIASxβ. Proc Natl Acad Sci.

[83] Kahyo T, Nishida T, Yasuda H (2001). Involvement of PIAS1 in the sumoylation of tumor suppressor p53. Mol Cell.

[84] Melchior F (2000). SUMO-nonclassical ubiquitin. Annu Rev Cell Dev Biol.

[85] Long J, Matsuura I, He D, Wang G, Shuai K, Liu F (2003). Repression of Smad transcriptional activity by PIASy, an inhibitor of activated STAT. Proc Natl Acad Sci.

[86] Lim CP, Cao X (2006). Structure, function, and regulation of STAT proteins. Mol BioSyst.

[87] Neel BG (1993). Structure and function of SH2-domain containing tyrosine phosphatases. Seminars in cell biology.

[88] Penninger JM, Irie-Sasaki J, Sasaki T, Oliveira-dos-Santos AJ (2001). CD45: new jobs for an old acquaintance. Nat Immunol.

[89] Andersen JN, Mortensen OH, Peters GH, Drake PG, Iversen LF, Olsen OH (2001). Structural and evolutionary relationships among protein tyrosine phosphatase domains. Mol Cell Biol.

[90] Singer CA, Lontay B, Unruh H, Halayko AJ, Gerthoffer WT (2011). Src mediates cytokine-stimulated gene expression in airway myocytes through ERK MAPK. Cell Communication and Signaling.

[91] Poole AW, Jones ML (2005). A SHPing tale: perspectives on the regulation of SHP-1 and SHP-2 tyrosine phosphatases by the C-terminal tail. Cell Signal.

[92] Klingmüller U, Lorenz U, Cantley LC, Neel BG, Lodish HF (1995). Specific recruitment of SH-PTP1 to the erythropoietin receptor causes inactivation of JAK2 and termination of proliferative signals. Cell.

[93] You M, Yu D-H, Feng G-S (1999). Shp-2 tyrosine phosphatase functions as a negative regulator of the interferon-stimulated Jak/STAT pathway. Mol Cell Biol.

[94] Myers MP, Andersen JN, Cheng A, Tremblay ML, Horvath CM, Parisien J-P (2001). TYK2 and JAK2 are substrates of protein-tyrosine phosphatase 1B. J Biol Chem.

[95] Pawson T, Scott JD (1997). Signaling through scaffold, anchoring, and adaptor proteins. Science.

[96] Velazquez L (2012). The Lnk adaptor protein: a key regulator of normal and pathological hematopoiesis. Arch Immunol Ther Exp.

[97] Murray PJ (2007). The JAK-STAT signaling pathway: input and output integration. J Immunol.

[98] Zabolotny JM, Bence-Hanulec KK, Stricker-Krongrad A, Haj F, Wang Y, Minokoshi Y (2002). PTP1B regulates leptin signal transduction in vivo. Dev Cell.

[99] Simoncic PD, Lee-Loy A, Barber DL, Tremblay ML, McGlade CJ (2002). The T cell protein tyrosine phosphatase is a negative regulator of janus family kinases 1 and 3. Curr Biol.

[100] Pickart CM (2001). Mechanisms underlying ubiquitination. Annu Rev Biochem.

[101] Ungureanu D, Saharinen P, Junttila I, Hilton DJ, Silvennoinen O (2002). Regulation of Jak2 through the ubiquitin-proteasome pathway involves phosphorylation of Jak2 on Y1007 and interaction with SOCS-1. Mol Cell Biol.

[102] Ikeda F (2015). Linear ubiquitination signals in adaptive immune responses. Immunol Rev.

[103] Swatek KN, Komander D (2016). Ubiquitin modifications. Cell Res.

[104] Yau R, Rape M (2016). The increasing complexity of the ubiquitin code. Nat Cell Biol.

[105] Daly C, Reich NC (1995). Characterization of specific DNA-binding factors activated by double-stranded RNA as positive regulators of interferon α/β-stimulated genes. J Biol Chem.

[106] Dao CT, Zhang D-E (2005). ISG15: a ubiquitin-like enigma. Front Biosci.

[107] Haas AL, Ahrens P, Bright P, Ankel H (1987). Interferon induces a 15-kilodalton protein exhibiting marked homology to ubiquitin. J Biol Chem.

[108] Kerscher O, Felberbaum R, Hochstrasser M (2006). Modification of proteins by ubiquitin and ubiquitin-like proteins. Annu Rev Cell Dev Biol.

[109] Malakhov MP, Kim KI, Malakhova OA, Jacobs BS, Borden EC, Zhang D-E (2003). High-throughput Immunoblotting ubiquitin-like protein isg15 modifies key regulators of signal transduction. J Biol Chem.

[110] Zhang D, Zhang D-E (2011). Interferon-stimulated gene 15 and the protein ISGylation system. J Interf Cytokine Res.

[111] Rogers RS, Horvath CM, Matunis MJ (2003). SUMO modification of STAT1 and its role in PIAS-mediated inhibition of gene activation. J Biol Chem.

[112] Chen Y, Jiao B, Yao M, Shi X, Zheng Z, Li S (2017). ISG12a inhibits HCV replication and potentiates the anti-HCV activity of IFN-α through activation of the Jak/STAT signaling pathway independent of autophagy and apoptosis. Virus Res.

[113] Jackson PK (2001). A new RING for SUMO: wrestling transcriptional responses into nuclear bodies with PIAS family E3 SUMO ligases. Genes Dev.

[114] Begitt A, Droescher M, Knobeloch K-P, Vinkemeier U (2011). SUMO conjugation of STAT1 protects cells from hyperresponsiveness to IFNγ. Blood.

[115] Van Nguyen T, Angkasekwinai P, Dou H, Lin F-M, Lu L-S, Cheng J (2012). SUMO-specific protease 1 is critical for early lymphoid development through regulation of STAT5 activation. Mol Cell.

[116] O'Brien KB, O'Shea JJ, Carter-Su C (2002). SH2-B family members differentially regulate JAK family tyrosine kinases. J Biol Chem.

[117] O'Shea JJ, Schwartz DM, Villarino AV, Gadina M, McInnes IB, Laurence A (2015). The JAK-STAT pathway: impact on human disease and therapeutic intervention*. Annu Rev Med.

[118] Ghoreschi K, Jesson MI, Li X, Lee JL, Ghosh S, Alsup JW (2011). Modulation of innate and adaptive immune responses by tofacitinib (CP-690,550). J Immunol.

[119] Tefferi A, Barbui T (2017). Polycythemia vera and essential thrombocythemia: 2017 update on diagnosis, risk-stratification, and management. Am J Hematol.

[120] Mazza P, Specchia G, Di Renzo N, Cascavilla N, Tarantini G, Capalbo SF (2017). Ruxolitinib–better prognostic impact in low-intermediate 1 risk score: evaluation of the ‘rete ematologica pugliese’(REP) in primary and secondary myelofibrosis. Leukemia & lymphoma.

[121] Ito M, Yamazaki S, Yamagami K, Kuno M, Morita Y, Okuma K (2017). A novel JAK inhibitor, peficitinib, demonstrates potent efficacy in a rat adjuvant-induced arthritis model. J Pharmacol Sci.

[122] Genovese M, Kremer J, Zamani O, Ludivico C, Krogulec M, Xie L (2015). OP0029 Baricitinib, an oral Janus Kinase (JAK) 1/JAK2 inhibitor, in patients with active rheumatoid arthritis (RA) and an inadequate response to TNF inhibitors: results of the phase 3 RA-beacon study. Ann Rheum Dis.

[123] Winthrop KL (2017). The emerging safety profile of JAK inhibitors in rheumatic disease. Nat Rev Rheumatol.

[124] Clark JD, Flanagan ME, Telliez J-B (2014). Discovery and development of Janus Kinase (JAK) inhibitors for inflammatory diseases: Miniperspective. J Med Chem.

[125] Thoma G, Nuninger F, Falchetto R, Hermes E, Tavares GA, Vangrevelinghe E (2010). Identification of a potent Janus kinase 3 inhibitor with high selectivity within the Janus kinase family. J Med Chem.

[126] Furumoto Y, Smith CK, Blanco L, Zhao W, Brooks SR, Thacker SG (2017). Tofacitinib ameliorates murine lupus and its associated vascular dysfunction. Arthritis Rheumatology.

[127] Fridman JS, Scherle PA, Collins R, Burn TC, Li Y, Li J (2010). Selective inhibition of JAK1 and JAK2 is efficacious in rodent models of arthritis: preclinical characterization of INCB028050. J Immunol.

[128] Wang JL, Cheng LP, Wang TC, Deng W, Wu FH. Molecular modeling study of CP-690550 derivatives as JAK3 kinase inhibitors through combined 3D-QSAR, molecular docking, and dynamics simulation techniques. J Mol Graphics Modell. 2017;72:178–86. 10.1016/j.jmgm.2016.12.020 28107751

[129] Cosgrove SB, Wren JA, Cleaver DM, Walsh KF, Follis SI, King VI (2013). A blinded, randomized, placebo-controlled trial of the efficacy and safety of the Janus kinase inhibitor oclacitinib (Apoquel®) in client-owned dogs with atopic dermatitis. Vet Dermatol.

[130] Little PR, King VL, Davis KR, Cosgrove SB, Stegemann MR (2015). A blinded, randomized clinical trial comparing the efficacy and safety of oclacitinib and ciclosporin for the control of atopic dermatitis in client-owned dogs. Vet Dermatol.

[131] Winton EF, Kota V (2017). Momelotinib in myelofibrosis: JAK1/2 inhibitor with a role in treating and understanding the anemia. Future Oncol.

[132] Mandal PK, Gao F, Lu Z, Ren Z, Ramesh R, Birtwistle JS (2011). Potent and selective phosphopeptide mimetic prodrugs targeted to the Src homology 2 (SH2) domain of signal transducer and activator of transcription 3. J Med Chem.

[133] Zhang W, Ma T, Li S, Yang Y, Guo J, Yu W (2017). Antagonizing STAT3 activation with benzo [b] thiophene 1, 1-dioxide based small molecules. Eur J Med Chem.

[134] Jia L, Song Q, Zhou C, Li X, Pi L, Ma X (2016). Dihydroartemisinin as a putative STAT3 inhibitor, suppresses the growth of head and neck squamous cell carcinoma by targeting Jak2/STAT3 signaling. PLoS One.

[135] Ahmad SF, Ansari MA, Nadeem A, Zoheir KM, Bakheet SA, Alsaad AM (2017). STA-21, a STAT-3 inhibitor, attenuates the development and progression of inflammation in collagen antibody-induced arthritis. Immunobiology.

[136] Kaltenecker D, Mueller KM, Benedikt P, Feiler U, Themanns M, Schlederer M (2017). Adipocyte STAT5 deficiency promotes adiposity and impairs lipid mobilisation in mice. Diabetologia.

[137] Timilshina M, Kang Y, Dahal I, You Z, Nam T-g, Kim K-J (2017). BJ-3105, a 6-Alkoxypyridin-3-ol analog, impairs T cell differentiation and prevents experimental autoimmune encephalomyelitis disease progression. PLoS One.

[138] Chen S, Bai Y, Li Z, Jia K, Jin Y, He B (2017). A betulinic acid derivative SH479 inhibits collagen-induced arthritis by modulating T cell differentiation and cytokine balance. Biochem Pharmacol.

[139] Zhao W, Jaganathan S, Turkson J (2010). A cell-permeable Stat3 SH2 domain mimetic inhibits Stat3 activation and induces antitumor cell effects in vitro. J Biol Chem.

